
JOURNAL INFORMATION
==============================
Journal ID: Wirtschaftsdienst

Wirtschaftsdienst

EISSN: 1613-978X

ARTICLE INFORMATION
==============================
PMCID: PMC9483306
DOI: 10.1007/s10273-022-3279-0
Article ID: 3279
Article version: 1
Subjects: Zeitgespräch

Zu wenig qualifizierte Arbeitskräfte für praktische Tätigkeiten

More and More Academics and Not Enough Skilled Workers

Brenke Karl kbrenke@diw.de

grid.8465.f 0000 0001 1931 3152 Deutsches Institut für Wirtschaftsforschung, Mohrenstraße 58, 10117 Berlin, Deutschland
Electronic publication date: 2022 Sep 16
Print publication date: 2022
Volume: 102
Issue: 9
First page: 677
Last page: 679
Copyright: © Der/die Autor:in 2022
License: Open Access: Dieser Artikel wird unter der Creative Commons Namensnennung 4.0 International Lizenz veröffentlicht (https://creativecommons.org/licenses/by/4.0/deed.de).
Open Access wird durch die ZBW — Leibniz-Informationszentrum Wirtschaft gefördert.
License URL: https://creativecommons.org/licenses/by/4.0/

Keywords: I28, J11, J20, O49
issue-copyright-statement: © The Author(s) 2022

==============================

Die wirtschaftlichen Einschränkungen seit Beginn der Coronapandemie waren erheblich – insbesondere infolge von staatlich verordneten Betriebseinstellungen. Es gab sie vor allem in Teilen der konsumnahen Dienstleistungen. In anderen Staaten wie China war auch das Verarbeitende Gewerbe betroffen, was weltweit zu Störungen in den Lieferketten bei Industriegütern führte. Nach dem starken Einbruch der Wirtschaftsleistung in der ersten Jahreshälfte 2020 ging es unter Schwankungen mit der Wertschöpfung wieder bergauf. Bei der Zahl der Erwerbstätigen wird der Vorseuchenwert mittlerweile sogar schon übertroffen – und zwar um 0,75 Mio. Inzwischen gibt es 42 Mio. Erwerbstätige – so viele wie noch nie in Deutschland. Die Kurzarbeit, mit der Arbeitseinschränkungen überbrückt wurden, ist stark zurückgefahren.

Hinter dem allgemeinen Aufschwung verbergen sich unterschiedliche Entwicklungen bei den einzelnen Gruppen von Erwerbstätigen. Getragen wurde der Beschäftigungsaufbau von der weitaus bedeutendsten Gruppe: den sozialversicherungspflichtig abhängig Beschäftigten. Im Aufwind sind auch wieder die Mini-Jobs. Allerdings ist hier die Zahl wie vor Corona noch nicht wieder erreicht. Unter den Selbstständigen, insbesondere unter den Solo-Selbständigen, verstärkte COVID-19 einen zuvor schon bestehenden Trend. Bereits ab 2012 nahm deren Zahl ab. Denn angesichts der damals guten Lage auf dem Arbeitsmarkt konnten nicht wenige Solo-Selbständige die unsichere und finanziell schwierige Selbständigkeit gegen eine sozialversicherungspflichtige Beschäftigung eintauschen. Mit der Pandemie ging die selbständige Beschäftigung dann stark zurück; eine deutliche Erholung zeigen die verfügbaren Daten nicht.

Wenn die Zahl der Beschäftigten wächst und sogar ein Rekordniveau erreicht wird, muss es nicht verwundern, dass hier und dort über einen Mangel an Arbeitskräften geklagt wird. Denn das ist der Chorgesang, der üblicherweise bei einem Beschäftigungsaufschwung angestimmt wird. Gleichwohl gibt es zunehmende Knappheiten, denn das Angebot an zusätzlich einstellbarem Personal hat zumindest nicht in gleichem Maße wie die Beschäftigung zugenommen. In dieser Hinsicht ist die Datenlage jedoch schwierig. In Deutschland wird üblicherweise auf die Zahl der registrierten Arbeitslosen gesehen. Bei diesem Indikator weiß man zum einen aber nicht, wie viele Arbeitslose wegen mangelnder Motivation oder gesundheitlicher Probleme dem Arbeitsmarkt überhaupt nicht zur Verfügung stehen. Zum anderen kann es Jobsuchende geben, die gar nicht als Arbeitslose registriert sind – beispielsweise weil sie keinen Anspruch auf Versicherungs- oder Sozialleistungen haben. Jedenfalls ist die Zahl der registrierten Arbeitslosen nach einem zeitweiligen kräftigen Anstieg deutlich gesunken. Das Niveau ist mit 2,4 Mio. jedoch nicht geringer als vor Beginn der Seuche – was allerdings auch mit den jüngsten Fluchtbewegungen aus der Ukraine zusammenhängt. International wird eher die Zahl der Erwerbslosen in den Blick genommen. Als erwerbslos gelten diejenigen Personen, die keinerlei bezahlter Tätigkeit nachgehen, die dem Arbeitsmarkt kurzfristig zur Verfügung stehen und aktiv einen Job suchen. Problematisch ist bei diesem Indikator vor allem, dass Unterbeschäftigung unzureichend erfasst wird. Die Zahl der Erwerbslosen ist nach einem coronabedingten Anstieg ebenfalls gesunken – und zwar stärker als die Zahl der registrierten Arbeitslosen. Inzwischen ist hier mit 1,3 Mio. der niedrigste Stand seit Einführung der monatlichen Erfassung dieser Statistik im Jahr 2005 erreicht worden.

## Engpässe bei mittlerer Qualifikation

Zur Ermittlung von Engpässen vergleicht die Bundesagentur für Arbeit die Zahl von Arbeitslosen mit der Zahl der ihr gemeldeten offenen Stellen. Auf dem Arbeitsmarkt gibt es allerdings ein ständiges Kommen und Gehen – und die offenen Stellen werden in der Regel mit Personen besetzt, die von einem in ein anderes Beschäftigungsverhältnis wechseln. Überdies gibt es nur die Zahlen über die bei den Arbeitsagenturen gemeldeten Vakanzen, die lediglich einen Teil des gesamtwirtschaftlichen Stellenangebots widerspiegeln. Und diese Teilmenge ist selektiv zusammengesetzt: Denn je höher die Ansprüche an die Qualifikation des gesuchten Personals sind, desto weniger werden bei der Stellensuche die Arbeitsagenturen eingeschaltet. Hierbei gibt es überdies Schwankungen je nach der Lage auf dem Arbeitsmarkt. Werden Arbeitskräfte knapper, suchen die Betriebe und andere Beschäftigungsstellen intensiver und melden ihre offenen Stellen häufiger der Arbeitsverwaltung. Bei den offenen Stellen wurde der Einbruch ab Frühjahr 2020 inzwischen überwunden und mit knapp 900.000 ein Wert erreicht, der etwas höher ist als zu der Zeit vor Corona. Ein Indiz für einen Arbeitskräftemangel in gesamtwirtschaftlichem Ausmaß ist das nicht.

Gleichwohl können Engpässe auf Teilarbeitsmärkten bestehen. Ein Vergleich der registrierten offenen Stellen für bestimmte Berufe oder Tätigkeiten mit den entsprechenden Arbeitslosen dürfte darauf vage Hinweise geben. So zeigt sich, dass es wohl nach wie vor schwerfällt, für all diejenigen Arbeitslosen, die lediglich für eine Helfertätigkeit infrage kommen, einen Job zu finden. Das gilt für alle Bereiche – auch für das Gesundheitswesen oder für die Altenpflege, wo es nach den verfügbaren Daten an qualifizierten Kräften mangelt. Bemerkenswert ist zudem, dass sich nur bei wenigen akademischen Berufen Engpässe andeuten. Ausnahme sind bestimmte Mediziner:innen, EDV-Berufe sowie Personen mit einer Ingenieurausbildung in den Bereichen Bau, Verkehrs-, Elektro- und Automatisierungstechnik, nicht jedoch in den „klassischen“ Ingenieurbereichen wie dem Maschinen- und dem Fahrzeugbau – hier haben die Arbeitslosenzahlen zuletzt sogar zugenommen. Viel häufiger scheint es ausgeprägte Knappheiten bei denjenigen Qualifikationen zu geben, die im Zuge einer Ausbildung im dualen System oder in einer Fachschule erworben werden. Neben Berufen in der Pflege und dem Gesundheitswesen trifft das für Teile der industriellen Fertigungsberufe (etwa für die Metallverarbeitung), die meisten Bauberufe, für Berufe im Eisenbahnverkehr, EDV-Berufe mittlerer Qualifikation sowie für Fachkräfte in der Gastronomie und für einige Büroberufe (etwa in der Steuerberatung) zu. Auffallend ist überdies, dass auf manchen solcher Berufsfelder, die mit Blick auf die Berufswahl besonders beliebt sind, die Zahl der Arbeitslosen diejenige der offenen Stellen weit übersteigt. Das gilt – unabhängig vom Grad des Berufsabschlusses – für die Tätigkeiten im Bereich Kommunikation, für künstlerische Jobs und auch für Tätigkeiten im Bereich der Geistes- und Sozialwissenschaften.

Der Blick auf die Berufe zeigt naturgemäß nur eine Momentaufnahme. Im Sommer 2022 ist auf dem Arbeitsmarkt der Effekt von Corona weitgehend überwunden. Nicht auszuschließen ist, dass alsbald die Seuche im Gewand einer neuen Mutationsvariante wieder das Wirtschaftsleben stört. Ganz sicher ist hingegen, dass sich die bereits grassierende Teuerung und die sich abzeichnende Krise bei der Energieversorgung infolge der Sanktionspolitik gegenüber Russland hemmend auf die Wirtschaftsleistung auswirken werden. Bei manchen der genannten Berufe könnten deshalb die Engpässe nur vorübergehender Natur sein.

## Zunehmende Alterung

Politisch relevant sind vor allem die grundlegenden den Arbeitsmarkt beeinflussenden Trends – und nicht so sehr die Wechselhaftigkeit des Wirtschaftsgeschehens in kurzer Frist. Von zentraler Bedeutung ist, dass sich die Bevölkerungsstruktur hin zu den Alten verschiebt. Zum einen steigt die Lebenserwartung. Im Besonderen kommt hinzu, dass die geburtenstarken Jahrgänge der Babyboomer-Generation das Rentenalter erreichen; dies wird sich noch etwa die nächsten zehn Jahre hinziehen. Zum anderen reicht – wenngleich es zuletzt eine leichte Besserung gab – die Geburtenhäufigkeit bei weitem nicht zur Reproduktion aus. Entsprechend werden die nachwachsenden Alterskohorten kleiner. Vor 20 Jahren gab es noch 1,5 Mio. mehr Kinder und Jugendliche als heute und vor 30 Jahren 1,7 Mio. mehr. Zwar zeigt sich seit geraumer Zeit eine vermehrte Erwerbsbeteiligung derjenigen Personen, die das gesetzliche Renteneintrittsalter erreicht haben. Das genügte aber nicht; seit Mitte des vergangenen Jahrzehnts schrumpft das bereits in Deutschland wohnende Erwerbspersonenpotenzial. Die Zuwanderungen hatten zwar in den letzten Jahren zugenommen, reichten aber nicht ganz aus, um den Effekt aufgrund der natürlichen Bevölkerungsbewegung auszugleichen.

Man muss weiterhin auf Zuwanderungen setzen. Allerdings wird man dadurch die Probleme nur abmildern können. Die Erwartungen sollten nicht zu hoch gesteckt werden. Denn viele andere Länder haben ähnliche demografische Probleme wie Deutschland. In jedem europäischen Land ist die Geburtenhäufigkeit zu gering – und zwar schon seit Jahrzehnten. In Europa beträgt die totale Fertilitätsrate 1,6 – ähnlich wie in Deutschland; zur Reproduktion sind aber etwas mehr als zwei Kinder pro Frau nötig. Auf einen solchen Wert kommen nur die Staaten der Subsahara, manche arabische Länder, Mittelamerika sowie Teile Süd- und Südostasiens. In den allermeisten Fällen handelt es sich dabei um weniger entwickelte Länder, was sich auch im Bildungswesen niederschlägt. Das spiegelt sich in den Problemen bei der Integration derjenigen Flüchtlinge wider, die insbesondere ab 2014 nach Deutschland kamen. Die bedeutendsten Herkunftsländer waren Syrien, Irak, Afghanistan, Iran, Pakistan, Eritrea, Somalia und Nigeria. Aus diesen Ländern stammten im April 477.000 der sozialversicherungspflichtig Beschäftigten, aber 574.000 der erwerbsfähigen Leistungsberechtigten für Hartz IV.

Aber selbst bei einer erfolgreicheren Zuwanderungspolitik wird man lediglich die Zunahme der Altenlast abschwächen können – darauf deuten die Daten der amtlichen Statistik zur „ersten mittelfristigen Bevölkerungsvorausberechnung 2021 bis 2035“ hin. Im Jahr 2020 kamen auf 100 Personen im Alter von 20 bis 66 Jahren 31,5 Personen ab 67 Jahren. Unter der Annahme, dass es zu hohen Zuwanderungszahlen kommt, werden es 37,3 Alte im Jahr 2030 sein – und im Jahr 2035 dann 41,3.

## Das Produktivitäts-Akademisierungs-Paradoxon

Der Königsweg wäre, mit einer steigenden Produktivität die Belastung durch die Alterung aufzufangen. Die tatsächliche Entwicklung geht jedoch in die andere Richtung. Denn der Tendenz nach nimmt in den westlichen Industriestaaten das Produktivitätswachstum ab; in Deutschland war es vor Corona sogar schon zum Stillstand gekommen. Dafür dürfte es mehrere Gründe geben. Für Deutschland lässt sich zeigen, dass der sektorale Wandel eine Rolle spielt: Einer wachsenden Bedeutung wenig produktiver Dienstleistungszweige steht die Abkehr von überdurchschnittlich produktiven Bereichen des produzierenden Gewerbes gegenüber. Das ist es aber nicht allein. Denn die Probleme bei der Produktivitätsentwicklung gehen mit einer zunehmenden Akademisierung des Arbeitskräftepotenzials einher. Da es keine rapide wachsende Akademikerarbeitslosigkeit gibt, lässt sich das Paradoxon nur damit erklären, dass sich der Einsatz der zusätzlichen Arbeitskräfte mit Hochschulabschluss nicht förderlich auf die Produktivitätsentwicklung auswirkt. Entsprechend ist in den angelsächsischen Ländern eine Debatte um eine zunehmende Bürokratisierung und die Ausbreitung unnützer Tätigkeiten aufgekommen. Für Deutschland lassen sich einige empirische Belege für eine entsprechende Verschiebung der Tätigkeitsstruktur finden.

Das mit großem Abstand beliebteste Studienfach in Deutschland ist die Betriebswirtschaftslehre. Darauf folgen Informatik und Jura. Theoretisch ist es schwer zu begründen, wie durch eine stetig zunehmende Zahl an Personen mit BWL- oder Juraabschluss der wirtschaftliche Wohlstand gemehrt werden könnte. Ähnliche Zweifel können auch mit Blick auf andere Hochschulfächer aufkommen – insbesondere dann, wenn unter dem Strich wenig an praktischem oder wissenschaftlichem Nutzen sichtbar wird.

Schon seit zehn Jahren beginnt in Deutschland mehr als die Hälfte eines Altersjahrgangs ein Studium. Da die nachwachsenden Generationen kleiner werden, gibt es entsprechend weniger junge Leute, die für eine Ausbildung im dualen System infrage kommen. Die Zeiten des Lehrstellenmangels sind inzwischen vorbei, nunmehr mangelt es an Auszubildenden. Im September 2021, dem Beginn des neuen Ausbildungsjahrs und Ende des üblichen Berichtsjahrs, kamen auf einen Ausbildungsplatz 0,83 Lehrstellensuchende. Und wenn es an Auszubildenden mangelt, fehlen bald die Fachkräfte. Wie angedeutet, trifft das wohl bereits jetzt schon für eine ganze Reihe von Berufen im Bereich der mittleren Qualifikation zu.

## Politische Folgerungen

Mit Blick auf die Demografie muss mehr auf eine kinderfreundliche Politik gesetzt werden und das Image der Familie ist zu stärken. Das ist eine langwierige Aufgabe. Vorrangig ist ebenfalls eine Debatte in Deutschland darüber, welche Berufe künftig überhaupt gebraucht werden. Die in der hiesigen Politik und auch in internationalen Institutionen verbreitete Vorstellung, dass ein immer größerer Teil der jungen Leute einen akademischen Abschluss haben sollte, passt wohl immer weniger zu den ökonomischen Erfordernissen. Das Land braucht nicht immer mehr Mundwerker, sondern eher mehr Handwerker bzw. generell mehr Personal für die praktischen Herausforderungen. Es gilt daher, einen Diskussionsprozess über die künftig erforderlichen Qualifikationen zu organisieren. Das könnte Aufgabe des Bundesbildungsministeriums sein – und eine Profilierungschance für diesen Fremdkörper im föderalen Gefüge. Die Zeit drängt, zumal eine Ausbildung dauert. Allein auf den Markt kann nicht gesetzt werden. Denn das Marktergebnis hängt immer von den Präferenzen und vom Verhalten der Menschen ab – und bei der Bürokratisierung spielt auch das Verlangen nach Macht und Einfluss eine Rolle. Auf jeden Fall wird es Widerstände bei einer Neuausrichtung der Berufsausbildung geben. Schließlich sollte sofort auf alles verzichtet werden, wodurch ein vorzeitiger Gang in den Ruhestand gefördert wird. Dazu gehört das Steuerprivileg bei der Altersteilzeit und der frühe und abschlagsfreie Rentenzugang für „langjährig Versicherte“. Statt all dem wird man wohl kaum über eine Anhebung des Rentenzugangsalters umhinkommen.

**Title:** *More and More Academics and Not Enough Skilled Workers*

All western countries are faced with the problem of a low birth rate and an increasingly aging population. The burden of old age requires accelerated productivity growth. Productivity growth is declining, however. In Germany, it has come to a standstill. At the same time, there is an increasing academisation of the labour force. There are several reasons for this paradox: sectoral change or bureaucratisation. A shortage of qualified blue collar workers is also a contributing factor.

Karl Brenke, Dipl.-Soziologe, ist Arbeitsmarktexperte am Deutschen Institut für Wirtschaftsforschung (DIW Berlin).

